# Ternary Arithmetic Logic Unit Design Utilizing Carbon Nanotube Field Effect Transistor (CNTFET) and Resistive Random Access Memory (RRAM)

**DOI:** 10.3390/mi12111288

**Published:** 2021-10-21

**Authors:** Furqan Zahoor, Fawnizu Azmadi Hussin, Farooq Ahmad Khanday, Mohamad Radzi Ahmad, Illani Mohd Nawi

**Affiliations:** 1 Electrical and Electronic Engineering Department, Universiti Teknologi PETRONAS, Seri Iskandar 32610, Malaysia; furqan_18000022@utp.edu.my (F.Z.); mohamadradzi.ahmad@utp.edu.my (M.R.A.); illani.nawi@utp.edu.my (I.M.N.); 2 Post Graduate Department of Electronics and Instrumentation Technology, University of Kashmir, Srinagar 190006, India; farooqkhanday@kashmiruniversity.ac.in

**Keywords:** multiple valued logic (MVL), resistive random access memory (RRAM), carbon nanotube field effect transistor (CNTFET), ternary logic systems, emerging technologies, innovation

## Abstract

Due to the difficulties associated with scaling of silicon transistors, various technologies beyond binary logic processing are actively being investigated. Ternary logic circuit implementation with carbon nanotube field effect transistors (CNTFETs) and resistive random access memory (RRAM) integration is considered as a possible technology option. CNTFETs are currently being preferred for implementing ternary circuits due to their desirable multiple threshold voltage and geometry-dependent properties, whereas the RRAM is used due to its multilevel cell capability which enables storage of multiple resistance states within a single cell. This article presents the 2-trit arithmetic logic unit (ALU) design using CNTFETs and RRAM as the design elements. The proposed ALU incorporates a transmission gate block, a function select block, and various ternary function processing modules. The ALU design optimization is achieved by introducing a controlled ternary adder–subtractor module instead of separate adder and subtractor circuits. The simulations are analyzed and validated using Synopsis HSPICE simulation software with standard 32 nm CNTFET technology under different operating conditions (supply voltages) to test the robustness of the designs. The simulation results indicate that the proposed CNTFET-RRAM integration enables the compact circuit realization with good robustness. Moreover, due to the addition of RRAM as circuit element, the proposed ALU has the advantage of non-volatility.

## 1. Introduction

The existing CMOS technology faces numerous critical issues in terms of high power dissipation, short channel effects, and reduced gate control when scaled to nanoscale dimensions. These reliability issues have the tendency to significantly degrade the system performance in the near future. The significant challenge facing the data-processing field is to provide effective technology capable of handling large amounts of data. Currently, semiconductor-based computation is the most used technology for such a task. This computation utilizes binary logic having two values (logic states) for its effective implementation. Several researchers over a period of time have pointed out various limitations of binary logic implementation, with the most significant one being that of the interconnection on chips as well as between the chips. In digital logic circuits, the main source of power dissipation are interconnects, and they occupy about 70% of the active logic elements which are mostly caused by placements of the digital logic components, complex routing, and electrical restrictions which are caused by the increasing number of connections [1]. The design of efficient digital systems becomes quite challenging when scaling down the feature size into a nano-meter regime owing to reliability and performance issues. Moreover, there is a limitation on the amount of binary data that can be transferred at a particular instant. Therefore, the tendency to consider alternate methods which do not focus on binary data for performing computation came to the fore. The most prominent approach to resolve these issues is to perform computation using multiple valued logic (MVL) instead of the binary logic implementation. MVL has received significant interest over the last couple of decades in the design of digital systems [2,3]. Since MVL utilizes more than two logic levels for the design implementation, it provides transmission of a larger amount of information over a single wire compared to the binary logic, resulting in higher design efficiency due to a lesser number of interconnects. On the basis of the logic levels used, MVL is subdivided into ternary (base = 3) or quaternary (base = 4) logic systems [4,5]. Ternary logic is the simplest and most effective implementation of MVL circuits, having significantly reduced complexity compared to that of the binary logic systems. In addition, ternary circuits can also help reduce the problems of pin-out and increase in interconnection density in the VLSI systems [6]. Depending on the external voltage, two implementations of the ternary logic exist, i.e., unbalanced ternary logic and balanced ternary logic [7]. In unbalanced representation, only positive voltages represented by logic levels {0, 1, 2} are used, whereas in the balanced representation, logic levels {−1, 0, 1} are used to represent the ternary numbers [8].

Considering various advantages of MVL designs, these circuits seem to be an obvious choice to replace the existing binary technologies, but with the integration of ICs and the modern technological evolution, the power and the speed enhancements of MVL designs do not seem adequate enough in the nano-scale regime [9]. Therefore, various emerging technologies and devices are being developed for effective implementation of MVL-based designs. MVL circuits were initially built using CMOS technology; additionally, some efforts were also made to combine binary and multi-valued blocks for CMOS-based designs [10,11]. Some efforts based on implementing MVL circuits utilizing quantum computing have also been made [12]. The computation capabilities in such systems are realized using quantum phenomena (quantum superposition and quantum entanglement) with quantum bit (qubit) as their basic building blocks. Moreover, Quantum Dot Cellular Automata (QCA) [13], and Single Electron Transistor (SET) [14] technologies for MVL design implementation were introduced of late. However, amongst these technologies, due to the remarkable characteristics of CNTFET, it is the preferred choice for MVL systems. Due to the resemblance of the CNTFET structure and the similarity in terms of its intrinsic properties with that of the conventional metal oxide semiconductor field effect transistor (MOSFET), CNTFET is the most viable nanodevice which can be utilized for implementing MVL-based designs as it eliminates the need for any major changes compared to the existing CMOS-based designs. Due to its unique 1D band structure, CNTFET has near-ballistic transport operation, thereby providing exceptional driving capability and lesser power dissipation [15]. Additionally, CNTFETs demonstrate better performance due to their greater carrier velocity, excellent carrier mobility and greater trans-conductance. Moreover, CNTFET is the preferred choice for effective realization of MVL-based circuits as multi-threshold design can be obtained easily by adjusting the carbon nanotube diameter, thereby altering the threshold voltage. This property is highly desirable for ternary logic design. Although CNT technology is the preferable choice for the MVL designs, it faces some drawbacks due to the lack of a defined method for the positioning of nanotubes, in addition to the complexity of the fabrication process. Despite these hindrances, significant research attempts are being undertaken to sort out these issues for effective technology implementation. While CNTFET production suitability is ongoing, the efforts to enhance performance of CNTFET designs can further be explored. Lately, graphene nanoribbon field effect transitors (GNRFETs) has emerged as a potential candidate for implementing MVL logic; however, CNTFET offers better $I_{ON}$/$I_{OFF}$ ratio, small subthreshold swing, and higher transconductance compared to GNRFET [16]. Furthermore, CNTs are immune to edge defects unlike nanoribbons, which is one of the the most important virtues of CNTs [17]. Although CNT technology is the preferable choice for the MVL designs, it faces some drawbacks due to the lack of a defined method for the positioning of nanotubes, in addition to the complexity of the fabrication process. The fabrication of CNTFETs is only being undertaken at limited academic or research facilities rather than the major commercial manufacturing facilities generating product wafers. This is because the initial technology transfer to CNTFET presents various hurdles as the methods involved in fabricating CNTFETs should comply with the strict compatibility demands of silicon-based commercial fabrication facilities. Despite these hindrances, significant research attempts are being undertaken to sort out these issues for effective technology implementation. Recently, Bishop et al. [18] demonstrated a solution-based CNT deposition technique to address these challenges and allow CNTFETs fabrication within the industrial facilities. While CNTFET production suitability is ongoing, the efforts to enhance the performance of CNTFET designs can further be explored.

Since demand for downscaling of electronic devices has become increasingly difficult to meet over the years, the need for new materials/mechanism-based devices compatible with the traditional CMOS has become quite realistic and attractive. Resistive random access memory (RRAM) has emerged as one of the effective solutions for addressing the continuous scaling down of electronic circuits. This is due to the fact that RRAM possesses many superior properties such as high storage density, non-volatility, good scalability, low ON-state resistance, and variable conductivity [19,20]. Taking into account the above-mentioned merits, RRAM can be considered as an effective technology candidate for intelligent computation with logic operation as an example. Thus, ternary systems can also be implemented utilizing RRAM as the design element. This is attributed to the fact that the RRAM device can effectively implement multiple logic values (two or more) without the need for extra hardware for realization of digital designs. Thus, the implementation of ternary logic utilizing RRAM needs to be explored further to enhance the system performance of the ternary logic systems. The basic structure of RRAM cells is a metal–insulator–metal (MIM) stack, whose electrical resistance can be switched by changing specific properties of the insulator layer on the application of external voltage pulse [21,22]. These bistable MIM cells switch back and forth between a high-resistance state (HRS) and low-resistance state (LRS) when an appropriate voltage is applied across the electrodes. This resistance switching is based on the formation and disruption of nanometer scale conductive filaments (CFs) [23]. For these CFs to be established initially, a first stage, referred to as forming operation, is required to drive the device into a conductive state referred to as LRS. During the reset operation, the CF is disrupted and the device is driven into HRS [24,25]. Subsequent recreations of the CF, which drive the device back to LRS, are known as set operations. This resistance shift between the HRS and LRS is effectively used for data storage in the form of ‘0’ and ‘1’ [26,27], respectively. In our work, we chose to implement our designs using RRAM as it enables high integration density, possesses simple structure, and can exhibit switching in the low-power regime [28]. Although RRAM-based logic implementation has gained significant interest and multiple design approaches have been successfully developed, all of these methods primarily focus on the investigation of binary logic implementation using RRAM. This technology implementation is still in its initial phases, as a significantly lesser amount of research has been undertaken for implementation of MVL-based circuits using RRAM.

In various digital applications, one of the core design components is the arithmetic and logic unit (ALU). An ALU that executes various logical as well as arithmetic operations is a critical design element of a digital computer [29]. To meet the increasing demands of high-performance processing systems, the effective design and realization of ALU is significantly important [30,31]. In this manuscript, we present 2-bit ternary ALU design utilizing CNTFET and RRAM device technologies. The addition of RRAM as the circuit element makes the ALU design non-volatile. This is the main advantage of the proposed architecture, and to date, no effort for the design of non-volatile ALU has been made. Additionally, efforts to reduce transistor count in the proposed design have been made by utilizing a negation of literals technique that enables reduction in the overall chip-area and cost. The proposed 2-bit ternary ALU has a single controlled ternary adder–subtractor unit, which eliminates the requirement of separate ternary adder and ternary subtractor modules in the design. Further, various ALU modules, i.e., ternary multiplier and ternary comparator, are implemented using CNTFET-RRAM technology, and these modules utilize binary gates in addition to the ternary gates. The presence of RRAM in the ternary design modules enables storage of logic states. This will ensure integration of logic computation and data storage into the proposed designs, which opens an emerging field of possibilities to explore intelligent computation systems.

The rest of the paper is presented as below. Section 2 provides a brief introduction of the CNTFETs. In Section 3, an overview of ternary logic is presented. Section 4 details the design methodology for various ternary combinational logic circuits and the architecture of ternary ALU. Simulation results obtained from HSPICE software and the comparative analysis is described in Section 5. Lastly, the conclusion is detailed in Section 6.

## 2. A Brief on Carbon Nanotube Field Effect Transistors (CNTFETs)

Recently, Carbon Nanotubes (CNTs) have come to the fore as most promising materials and are being considered for a wide variety of applications ranging from large-scale structure to nanometer-scale electronics because of their superior mechanical and physical properties [32,33]. CNTs were discovered accidentally by Japanese physicist S. lijima [34] in 1991 when performing experiments on carbonium. CNTs are basically nano-scaled tubes formed by the rolled sheets of graphite and possess high tensile strength, high electrical conductivity and excellent chemical stability [35]. CNTs are the smallest-scale nano-materials that can be observed only by a transmission electron microscope (TEM) [36,37]. CNTs are classified as either single-walled (SWCNT), which consist of a single nanotube, or multi-walled (MWCNT), which are made up of multiple nanotubes with inter-layer spacing of 0.34 nm. The CNTFET electrical characteristics are dependent on chirality vector specified by the integer pair (n, m). The chiral vector indices indicate the graphene sheet rolling direction. For example, if n − m = 3k, where ‘k’ is an integer, SWCNT has metallic features, otherwise the SWCNT is semiconducting. Metallic SWCNTs are used as on-chip interconnects, whereas the semiconducting ones are used as the channel of CNT-based devices [18,38].

**Figure 1:**
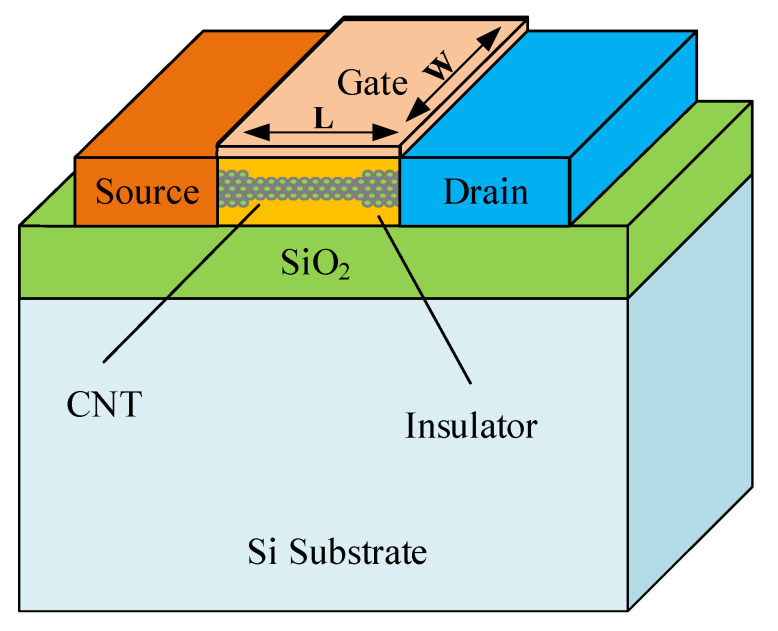
Device structure of a Carbon Nanotube Field Effect Transistor (CNTFET).

A typical CNTFET device structure is depicted in Figure 1. The CNT in the device performs as a channel of the transistor which can be modified by a gate. The drain and source terminals of CNTFET are heavily doped, whereas the gate region can be non-doped. The CNTFET gate width is determined by number of SWCNTs that are placed adjacent to each other [39]. The distance between the axes of two adjacent SWCNTs is referred to as pitch value, whereas the CNTFET gate width is determined from Equation (1) as [40].

(1) $$W_{gate}\approx Max\left(W_{min},M\times Pitch\right)$$

where $W_{min}$ is the minimum width of the CNTFET gate and M is the number of SWCNTs placed under the transistor gate.

To calculate the diameter of the CNT, we make use of Equation (2) and (3) as [41].

(2) $$D_{CNT}=\frac{\sqrt{3}a_{0}}{\pi }\sqrt{n^{2}+m^{2}+nm}$$

(3) $$\approx 0.0783\sqrt{n^{2}+m^{2}+nm}$$

where $a_{0}$ = 0.142 nm is the distance between the neighboring carbon atoms and n, m are the chirality vectors specifying the roll orientation of CNT.

The CNTFET device structure, its working operation, and the current–voltage (I-V) characteristics are similar to that of the traditional MOSFET. When the gate voltage is less than the threshold voltage (minimum voltage that must be applied to turn transistor ‘ON’), the drain current is almost zero, and increasing the gate voltage more than the transistor threshold voltage leads to an increase in drain current. Since the threshold voltage of CNTFET depends on the diameter of the nanotube, the threshold voltage of CNTFET [41] is expressed in Equation (4) as:

(4) $$V_{th}\approx \frac{E_{g}}{2e}=\frac{\sqrt{3}}{3}\frac{a}{e}\frac{V_{\pi }}{D_{CNT}}\approx \frac{0.436}{D_{CNT}}$$

where *e* is the electron charge, $E_{g}$ represents bandgap, *a* = 2.49 Å is the carbon atom distance, $V_{\pi }$ = 3.033 eV is the carbon π-π bond energy in the tight bonding model.

Thus, for any circuit implementation, multiple threshold voltages are obtained simply by changing the nanotube diameter, which is dependent on the the chirality vector (n, m). Thus, by changing the chirality vector or the diameter of the CNT, it is possible to control the threshold voltage of CNTFET [42]. This makes CNTFET a favorable prospect for the implementation of ternary-logic-based designs in contrast to the CMOS devices.

## 3. Overview of Ternary Logic

Ternary logic is one of the most widely used sub-categories of MVL, which comprises three different logic levels [43,44]. The addition of a third logic state to the existing binary logic results in ternary logic functions. In the case of ternary logic systems, voltage levels 0 V, $\frac{V_{dd}}{2}$, and $V_{dd}$ represent logic values ‘0’, ‘1’, and ‘2’, respectively [45,46].

Let us assume that the ternary values representing false, undefined, and true conditions are 0, 1, and 2, respectively. Any ternary function of n variables ($Y_{1}$, $Y_{2}$ . . . $Y_{n}$) is defined as a logic function mapping from ${\left\{0,1,2\right\}}^{n}$ to {0, 1, 2}. The fundamental ternary logic operations are defined in Equation (5) as [47].

$$\bar{Y_{i}}=2-Y_{i}$$

$$Y_{i}\;\cdot \;Y_{j}=min\left\{Y_{i},Y_{j}\right\}$$

(5) $$Y_{i}+Y_{j}=max\left\{Y_{i},Y_{j}\right\}$$

where $Y_{i}$, $Y_{j}$ε{0,1,2}, and ‘-’ represent the arithmetic subtraction. ‘$\bar{Y_{i}}$’ refers to the logical NOT operation, whereas ‘$Y_{i}\;\cdot \;Y_{j}$’ and ‘$Y_{i}+Y_{j}$’ represent logical AND and logical OR operations, respectively. Table 1 shows the logic symbols that we assume for ternary logic design.

For effective design of digital systems, the implementation of fundamental gates, i.e., the inverter, the NOR gate, and the NAND gate, is quite important. The fundamental ternary gates are designed according to the convention defined by Equation (5).

Ternary inverters are categorized as: standard ternary inverter (STI), positive ternary inverter (PTI), and negative ternary inverter (NTI). The ternary inverters (STI, PTI, NTI) truth table is shown below in Table 2. STI is basically a ternary NOT function that returns output logic ‘0’ for input state logic ‘2’ and vice versa, whereas the logic state ‘1’ remains unchanged. For NTI, inputs of logic ‘2’ and logic ‘0’ are inverted, and the same happens for the STI function, but NTI returns the output of logic ‘0’ for the input state logic ‘1’. For the case of PTI, inputs of logic ‘2’ and logic ‘0’ are inverted, but PTI returns an output of logic ‘2’ for the input state logic ‘1’. The STI, PTI, and NTI functions are given by Equations (6), (26), and (8) as:

(6) $$Z_{STI}=f_{0}\left(y\right)=2-y$$

(7) $$Z_{PTI}=f_{1}\left(y\right)=\left\{\begin{array}{cc} 2, & if\;y\neq 2\\ 0, & if\;y=2 \end{array}\right.$$

(8) $$Z_{NTI}=f_{2}\left(y\right)=\left\{\begin{array}{cc} 2, & if\;y=0\\ 0, & if\;y\neq 0 \end{array}\right.$$

The ternary NAND and ternary NOR functions for the inputs $Y_{1}$ and $Y_{2}$ are defined by Equation (9) and Equation (10), respectively.

(9) $$Z_{NAND}=\bar{min\left\{Y_{1},Y_{2}\right\}}$$

(10) $$Z_{NOR}=\bar{max\left\{Y_{1},Y_{2}\right\}}$$

The truth table for the ternary NAND and NOR gates is shown in Table 3.

Additionally, to reduce the number of components required for representing combinational logic functions, the negation of literals technique is utilized. The negation of literals ($Z_{i}$), given by Equations (11)–(15), is quite useful in reducing the number of ternary gates.

(11) $$NEG\left(Z_{i}\right)=\{\begin{array}{cc} 0, & \mathrm{if}Z=i\\ 2, & \mathrm{if}Z\neq i \end{array}$$

(12) $$Z_{2}=\bar{Z_{01}}\;\&\;Z_{01}=\bar{Z_{2}}$$

(13) $$Z_{1}=\bar{Z_{02}}\;\&\;Z_{02}=\bar{Z_{1}}$$

(14) $$Z_{0}=\bar{Z_{12}}\;\&\;Z_{12}=\bar{Z_{0}}$$

(15) $$\bar{0_{}}=2\;\;\bar{2_{}}\;=\;0\;$$

## 4. Proposed CNTFET-RRAM Ternary Arithmetic Logic Unit (ALU) Architecture and Functionality

**Figure 2:**
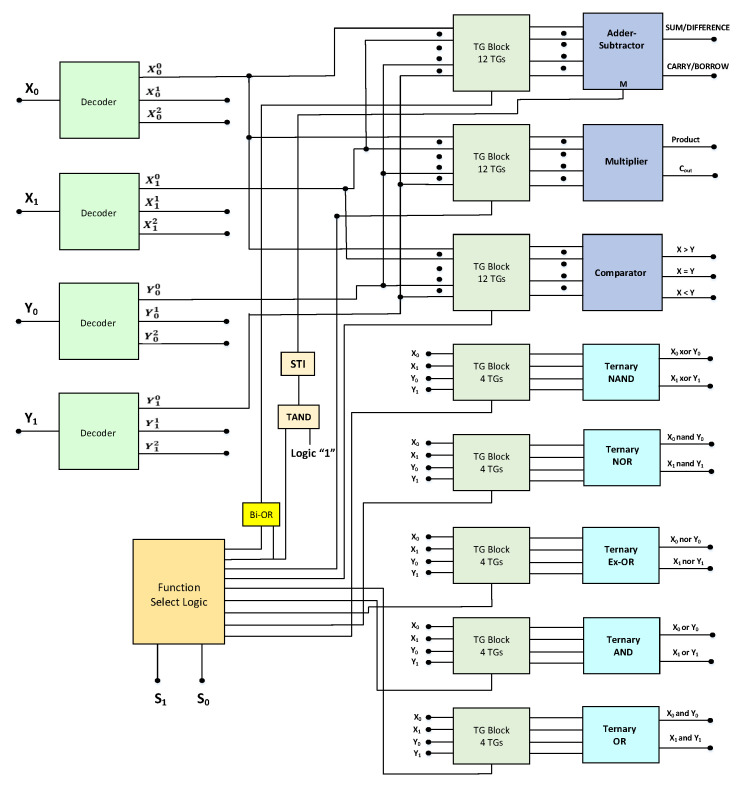
Proposed architecture of 2-trit ternary ALU.

One of the most significant building blocks of every processor is the ALU, which has the capability to perform both the arithmetic and logic operations. For the case of ternary ALU, voltage levels 0 V, $\frac{V_{dd}}{2}$, and $V_{dd}$ represent logic values ‘0’, ‘1’, and ‘2’, respectively. A block diagram depicting the architecture of the ternary ALU is shown in Figure 2. The ALU performs four arithmetic and five logic operations. The arithmetic operations include ternary addition, subtraction, multiplication, and comparison, whereas the logical operations performed are the ternary NAND, NOR, Ex-OR, AND, and OR.

**Figure 3:**
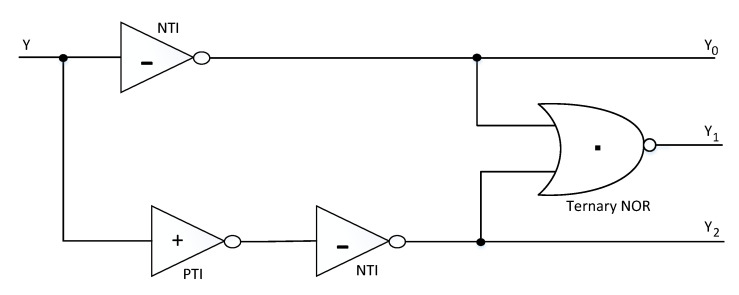
Schematic of ternary decoder [11].

From the block diagram, the main components of ternary ALU are the decoders, function select logic, transmission gate (TG) block, and various functional processing modules. This architecture is similar to the ternary ALU designs presented in [29,30,31]. A digit in binary logic is called a bit, so in ternary logic, we term it as trit. The ternary ALU block takes two 2-trit numbers as inputs ( $X_{0}$$X_{1}$ and $Y_{0}$$Y_{1}$ ) and produces a 2-trit output. The ternary decoder generates three unary functions for each of the inputs (X and Y), which are then further utilized for ternary function implementation. The schematic of ternary decoder is depicted in Figure 3, and the response of ternary decoder to an input y is given by Equation (16) as [11]:

(16) $$Y_{n}=\{\begin{array}{cc} 2, & \mathrm{if}Y=n\\ 0, & \mathrm{if}Y\neq n \end{array}$$

where n can take logic values 0, 1, or 2.

**Figure 4:**
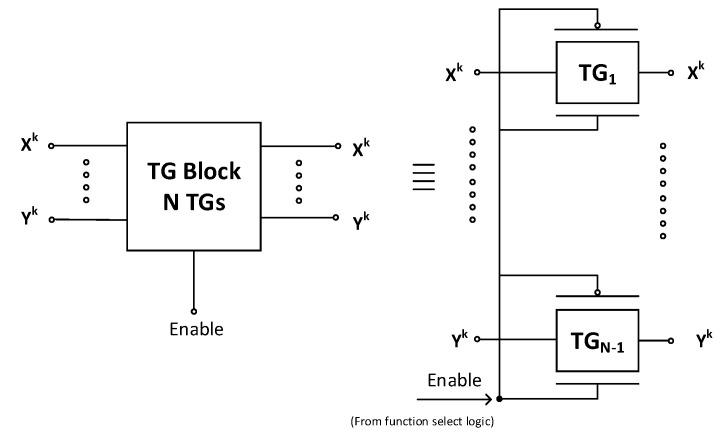
Transmission gate (TG) block logic diagram [29].

The TG block logic schematic is shown in Figure 4. The TG block comprises the number of transmission gates which connect the inputs $X_{0}$$X_{1}$ and $Y_{0}$$Y_{1}$ to various arithmetic and logical modules to perform the desired operation. The parallel connection of PCNTFET and NCNTFET is used for the implementation of the transmission gate.

**Figure 5:**
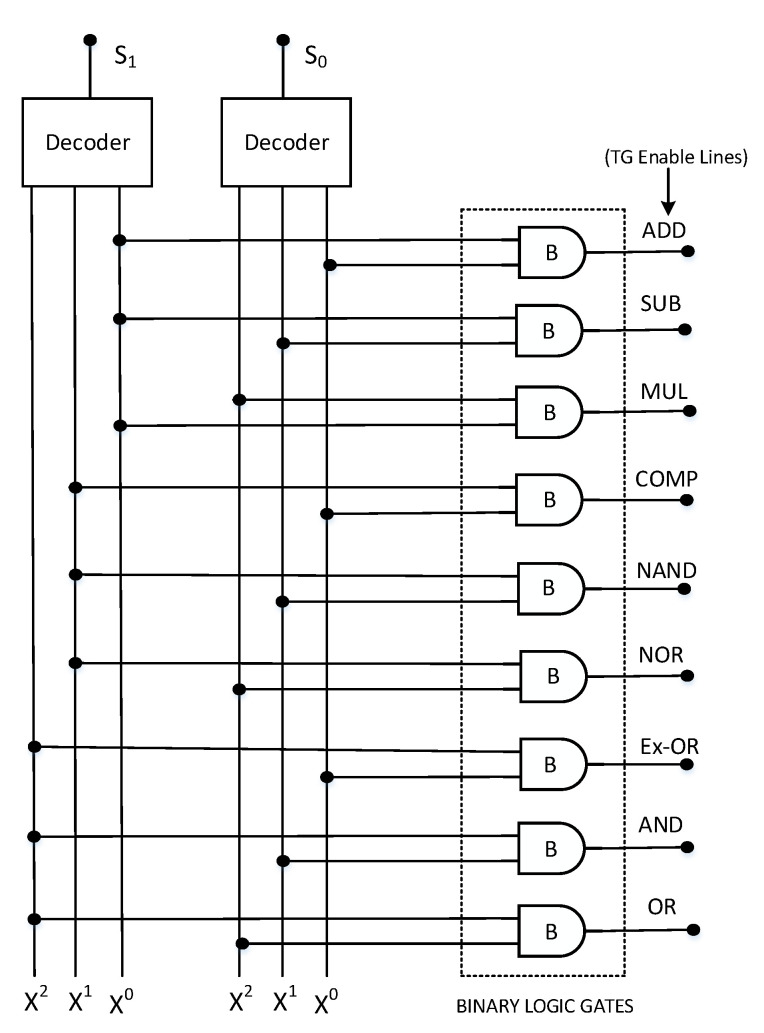
Schematic diagram of function select logic block [30].

The activation of the particular TG block depends on the select line $S_{0}$ and $S_{1}$ values, thus connecting the inputs to the corresponding functional module. The circuit schematic of the function select block consisting of decoders and array of binary AND gates is depicted in Figure 5. The function select block consists of two input select lines $S_{0}$$S_{1}$, producing nine different output combinations that act as enable lines. The output of the ternary ALU is determined by the value of the select lines $S_{0}$$S_{1}$ of the function select logic block, and these select lines determine the operation that the ternary ALU performs on the inputs. The proposed ternary ALU truth table is shown in Table 4. The arithmetic or the logic operation to be performed is determined by the function select logic block, whereas the TG block provides the necessary inputs to various processing modules to perform the selected operation. The addition and the subtraction operation is performed by a single adder–subtractor module, depending upon the mode control signal ‘M’. For $S_{0}$ = 0 and $S_{1}$ = 0, the first transmission line will be enabled, while the remaining transmission lines will be disabled. The output of the binary OR gate will be high and this will enable the TG block corresponding to the adder–subtractor module. The value of the mode control signal ‘M’ will be logic ‘2’. This is achieved by utilizing the ternary NAND and STI circuit as depicted in the architecture of ALU. Thus, the ALU performs the addition operation for this case. Similarly, For $S_{0}$ = 0 and $S_{1}$ = 1, the second transmission line will be enabled, while the remaining transmission lines will be disabled. The output of the binary OR gate will be high, and this will enable the TG block corresponding to the adder–subtractor module. The value of the mode control signal ‘M’ will be logic ‘1’. This is achieved by utilizing the ternary NAND and STI circuit as depicted in the architecture of ALU. Thus, the ALU performs the subtraction operation for this case. Similarly, various arithmetic and logic operations are performed depending upon the value of the select lines of the function logic block. The design of various arithmetic modules such as the adder–subtractor block, multiplier block, and comparator block along with various logical blocks such as ternary NAND, NOR, Ex-OR, AND, and OR is presented in the section below.

### 4.1. Logical Modules (Ternay NAND, Ternary NOR, Ternary Ex-OR)

**Figure 6:**
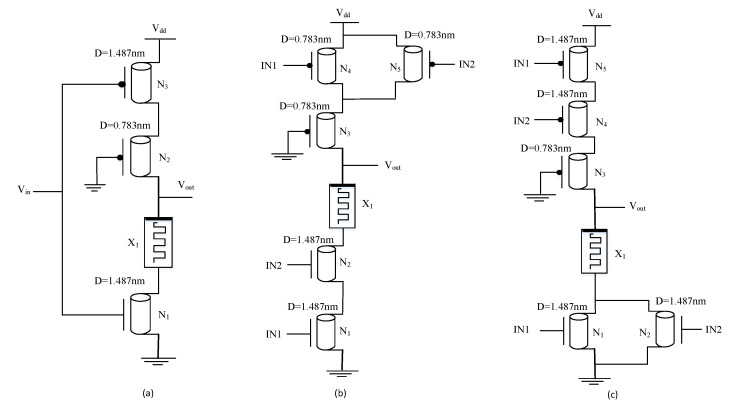
Circuit diagram of (**a**) STI, (**b**) ternary NAND, (**c**) ternary NOR [11].

For improvement in performance parameters, particularly in terms of low power consumption, CNTFET-RRAM ternary logic gates are implemented as they avoid using large resistor values in the design of ternary gates. For the design of digital systems, the basic gates are inverters, and the universal gates, i.e., NAND gates and NOR gates. The CNTFET-RRAM-based STI described previously in [11] is depicted in Figure 6a. The schematic of STI consists of two CNTFETs ($N_{1}$, $N_{3}$), a grounded gate p-type CNTFET ($N_{2}$), and an RRAM device ($X_{1}$). The CNTs $N_{1}$, $N_{2}$, and $N_{3}$ have chirality (19, 0), (10, 0), and (19, 0), respectively. From Equation (2), the diameters of $N_{1}$, $N_{2}$, and $N_{3}$ are 1.487 nm, 0.783 nm, and 1.487 nm, respectively. Therefore, from Equation (4), the threshold voltages of $N_{1}$, $N_{2}$, and $N_{3}$ are 0.293 V, −0.557 V, and −0.293 V, respectively. The number of tubes used for $N_{1}$ and $N_{2}$ is 3, while for $N_{2}$ it is 1. The CNTFET-RRAM ternary NAND and NOR gate schematic is depicted in Figure 6b,c, respectively. The CNT chirality used in $N_{1}$, $N_{2}$, $N_{4}$, $N_{5}$ is (19, 0), while for $N_{3}$ it is (10, 0) for both the ternary NAND and NOR gates. The number of tubes used for $N_{1}$, $N_{2}$, $N_{4}$, $N_{5}$ is 3, while for $N_{3}$ is 1.

**Figure 7:**
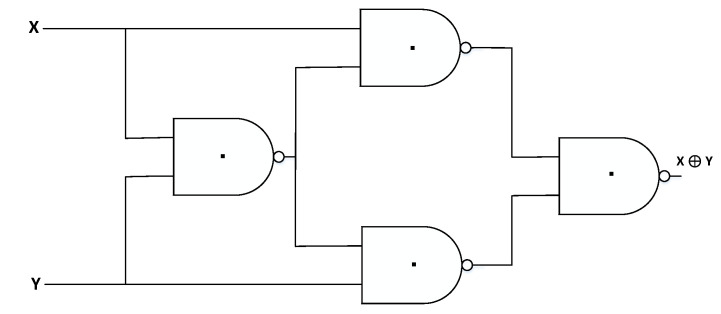
CNTFET-RRAM ternary Ex-OR gate.

The ternary CNTFET-RRAM-based Ex-OR gate is designed utilizing the universal property of NAND gates. The circuit schematic of ternary Ex-OR gates using CNTFET-RRAM ternary NAND gates is shown in Figure 7.

### 4.2. Design of Ternary Adder–Subtractor Module

**Figure 8:**
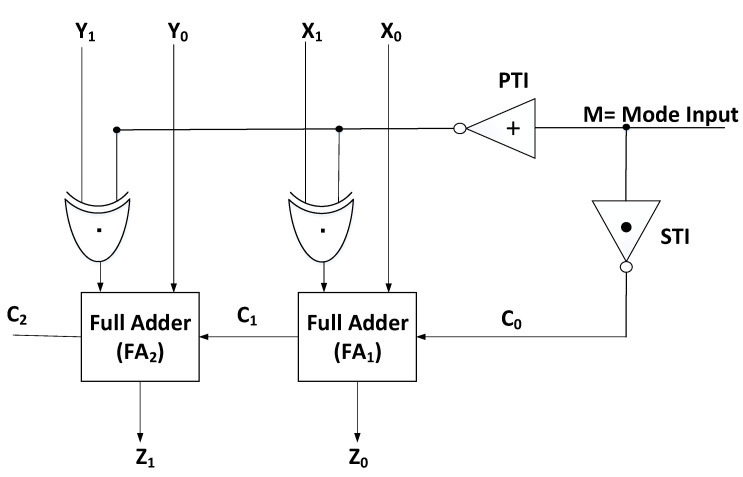
Design implementation of CNTFET-RRAM ternary adder–subtractor.

The proposed adder–subtractor block utilizing CNTFET-RRAM ternary gates for implementation effectively carries out the addition and subtraction operation on 2-trit ternary numbers. The proposed adder–subtractor module combines the functionality of addition and subtraction into a single circuit. The circuit schematic of the proposed CNTFET-RRAM ternary adder–subtractor is depicted in Figure 8. The adder–subtractor module is implemented using single STI and PTI circuits, two ternary full adders together with two ternary Ex-OR gates. The adder–subtractor module performs both as an adder and subtractor with the help of a mode control input ‘M’.

When Mode input M = 2, the addition function is performed by the adder–subtractor circuit. When input M = 2 is applied on the input of the PTI, it gets converted to 0, and this PTI output is applied to one of the inputs of the first Ex-OR gate, with the other input as $X_{1}$. The output of the Ex-OR gate in this case will be the same as input $X_{1}$. This output signal $X_{1}$ from the EX-OR gate will serve as one of the inputs of the first full adder ($X_{1}$), with another input being $X_{0}$. Moreover, since M = 2, the carry signal ($C_{0}$) from the STI to the full adder will be 0. Thus, the circuit behaves as an adder, performing the addition function of X and Y inputs.

When Mode input M = 1, the subtraction function is performed by the adder–subtractor circuit. The ternary subtraction X-Y is performed by taking 3’s complement of Y and adding it to X. To perform subtraction function, the carry signal ($C_{0}$) from the STI to the full adder will be 1. When input M = 1 is applied on the input of the PTI, it gets converted to 0, this PTI output is applied to one of the inputs of the first Ex-OR gate, and at the output of the Ex-OR gate, we obtain the 1’s complement of Y. To obtain 3’s complement of Y, 1 is added to the full adder using Mode input M = 1 obtained from STI attached. Thus, the circuit performs the function X + 3’s complement of Y. In the case of signed numbers, the overflow is detected by the carry bit ($C_{2}$). For unsigned binary numbers, carry bit ($C_{2}$) is used to determine a carry after the addition or borrow after subtraction. In the case of subtraction, the signed numbers are identified if the $C_{2}$ bit is ‘1’, indicating an overflow. For addition, we simply discard the overflow bit, whereas for subtraction, the overflow bit indicates that the result is a signed number and its magnitude is determined by taking 3’s complement of the obtained result.

**Figure 9:**
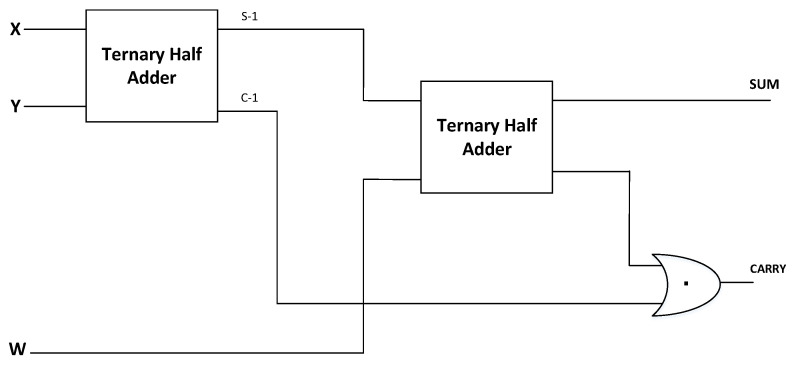
Block diagram of CNTFET-RRAM ternary full adder.

**Figure 10:**
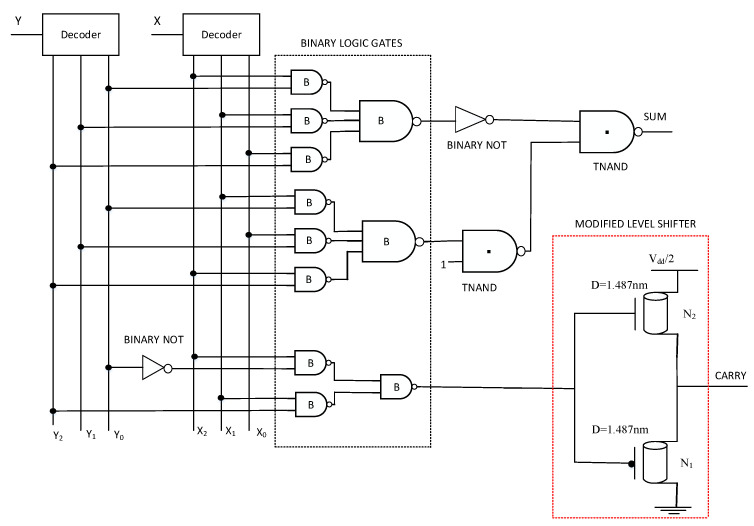
Circuit schematic of CNTFET-RRAM ternary half adder [40].

The ternary full adder designed using ternary gates is implemented using two CNTFET-RRAM ternary half adders and a ternary OR logic gate, as is shown in Figure 9. The ternary half adder circuit diagram shown in Figure 10 has two inputs ‘X’ and ‘Y’ and two outputs ‘SUM’ and ‘CARRY’ based on Equation (17) as [40].

$$SUM=X_{0}Y_{2}+X_{1}Y_{1}+X_{2}Y_{0}=1\;\cdot \;\left(X_{0}Y_{1}+X_{1}Y_{0}+X_{2}Y_{2}\right)$$

(17) $$CARRY=1\;\cdot \;(X_{2}\left[Y_{1}+Y_{2}\right]+X_{1}Y_{2})$$

The equation for CARRY, due to the negation of literals [47], is given in (18) as:

(18) $$CARRY=1\;\cdot \;\left(X_{2}Y_{12}+X_{1}Y_{2}\right)=1\;\cdot \;\left(X_{2}\bar{Y_{0}}+X_{1}Y_{2}\right)$$

The SUM and CARRY functions specified in Equations (17) and (18) are implemented using the binary gates along with the CNTFET-RRAM ternary gates. A modified level shifter (LS) circuit shown in Figure 10 is implemented to obtain a logic function as [40]:

(19) $$Out=\{\begin{array}{cc} 1, & \;\;\;\mathrm{if}\;\mathrm{in}=1,2\\ 0, & \mathrm{if}\;\mathrm{in}=0 \end{array}$$

### 4.3. Ternary Multiplier Module

**Figure 11:**
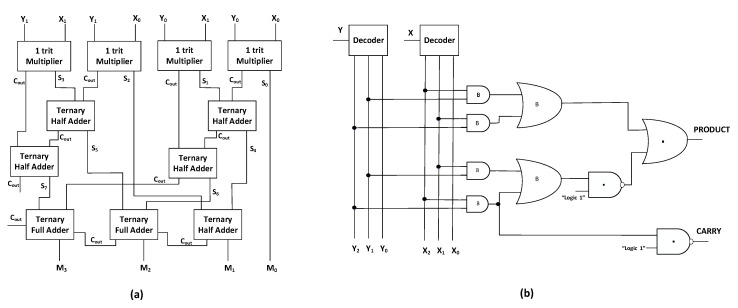
(**a**) Block diagram of 2-trit multiplier [49]. (**b**) Circuit schematic of 1-trit CNTFET-RRAM ternary multiplier [11].

This module performs a 2-trit ternary numbers multiplication operation, generating the product and the carry outputs [48]. The block diagram implementation of 2-trit multiplier is shown in Figure 11a. It makes use of 1-trit multiplier, half adder, and full adder blocks to realize the 2-trit multiplier functionality. The proposed multiplier architecture resembles the multiplier of Dhande and Ingole [49]. The ternary half adder and ternary full adder modules are implemented using the CNTFET-RRAM ternary logic gates using the design methodology discussed in the previous section. The schematic of 1-trit multiplier is shown in Figure 11b. It achieves the desired functionality using combination of binary logic gates and CNTFET-RRAM ternary logic gates. The ternary multiplier output equations are obtained as [11]:

(20) $$Product=X_{2}Y_{1}+X_{1}Y_{2}+1\;\cdot \;\left(X_{1}Y_{1}+X_{2}Y_{2}\right)$$

(21) $$CARRY=1\;\cdot \;(X_{2}Y_{2})$$

### 4.4. Ternary Comparator Module

**Figure 12:**
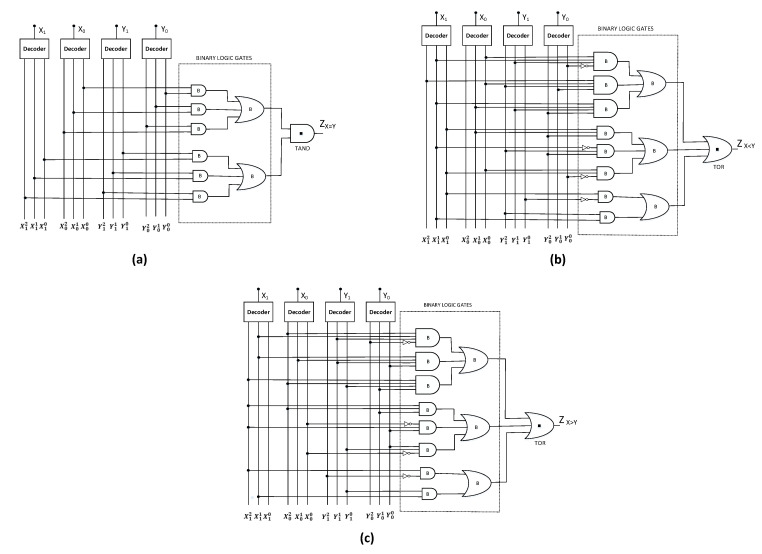
Schematic of ternary comparator. (**a**) X = Y, (**b**) X < Y, (**c**) X > Y.

A comparator is a combinational circuit that performs a comparison among two ternary numbers and determines whether the number is greater than, equal to, or less than the other given number [50]. The circuit schematic of 2-bit ternary comparator implemented using CNTFET-RRAM ternary logic gates is depicted in Figure 12.

For the case of ternary equality comparator shown in Figure 12a, the magnitudes of two numbers X ($X_{1}$$X_{0}$) and Y ($Y_{1}$$Y_{0}$) are compared, and the output $Z_{X=Y}$ is set as logic “2” if and only if X = Y, otherwise logic “0” is obtained as the output. The output equation for this case is as presented in Equation (22) as [47]:

(22) $$\begin{array}{cc} Z_{X=Y}= & X_{0}^{0}X_{1}^{0}Y_{0}^{0}Y_{1}^{0}+X_{0}^{1}X_{1}^{0}Y_{0}^{1}Y_{1}^{0}+X_{0}^{2}X_{1}^{0}Y_{0}^{2}Y_{1}^{0}+X_{0}^{0}X_{1}^{1}Y_{0}^{0}Y_{1}^{1}+X_{0}^{1}X_{1}^{1}Y_{0}^{1}Y_{1}^{1}\\ \; & +X_{0}^{2}X_{1}^{1}Y_{0}^{2}Y_{1}^{1}+X_{0}^{0}X_{1}^{2}Y_{0}^{0}Y_{1}^{2}+X_{0}^{1}X_{1}^{2}Y_{0}^{1}Y_{1}^{2}+X_{0}^{2}X_{1}^{2}Y_{0}^{2}Y_{1}^{2}\\ \; \end{array}$$

(23) $$Z_{X=Y}=\left[X_{0}^{0}Y_{0}^{0}+X_{0}^{1}Y_{0}^{1}+X_{0}^{2}Y_{0}^{2}\right]\;\cdot \;\left[X_{1}^{0}Y_{1}^{0}+X_{1}^{1}Y_{1}^{1}+X_{1}^{2}Y_{1}^{2}\right]$$

For ternary less than comparator depicted in Figure 12b, the output $Z_{X<Y}$ sets to logic ‘2’ if and only if X < Y, otherwise logic ‘0’ is obtained as the output on comparing magnitudes of two numbers X ($X_{1}$$X_{0}$) and Y ($Y_{1}$$Y_{0}$). The output equation for this case is as presented in Equation (24) [47]:

(24) $$\begin{array}{cc} Z_{X<Y}= & X_{0}^{0}X_{1}^{1}Y_{0}^{1}Y_{1}^{1}+X_{0}^{0}X_{1}^{2}Y_{0}^{1}Y_{1}^{2}+X_{0}^{0}X_{1}^{1}Y_{0}^{2}Y_{1}^{1}+X_{0}^{1}X_{1}^{1}Y_{0}^{2}Y_{1}^{1}+2X_{1}^{0}Y_{1}^{1}+2X_{1}^{0}Y_{1}^{2}+2X_{1}^{1}Y_{1}^{2}\\ & +2Y_{0}^{2}Y_{1}^{2}\left[X_{0}^{0}+X_{0}^{1}\right]+X_{0}^{0}X_{1}^{0}Y_{0}^{1}+2X_{1}^{0}Y_{0}^{2}\left[X_{0}^{0}+X_{0}^{1}\right] \end{array}$$

The negation of literals approach reduces the output $Z_{X<Y}$ to:

(25) $$\begin{array}{cc} Z_{X<Y}= & X_{1}^{0}\bar{Y_{1}^{0}}+X_{1}^{1}Y_{1}^{2}+X_{0}^{0}X_{1}^{0}\bar{Y_{0}^{0}}+\bar{X_{0}^{2}}Y_{0}^{2}Y_{1}^{2}+X_{0}^{1}X_{1}^{0}Y_{0}^{2}\\ \; & +X_{0}^{0}X_{1}^{1}\bar{Y_{0}^{0}}Y_{1}^{1}+X_{0}^{1}X_{1}^{1}Y_{0}^{2}Y_{1}^{1}+X_{0}^{0}X_{1}^{2}Y_{0}^{1}Y_{1}^{2}\\ \; \end{array}$$

Similarly, in the ternary greater than comparator depicted in Figure 12c, the output $Z_{X>Y}$ sets to logic ‘2’ if and only if X > Y, otherwise logic ‘0’ is obtained as the output on comparing magnitudes of two numbers X ($X_{1}$$X_{0}$) and Y ($Y_{1}$$Y_{0}$). The output is given in Equation (25) [47]:

(26) $$\begin{array}{cc} Z_{X>Y}= & X_{0}^{2}X_{1}^{1}Y_{0}^{0}Y_{1}^{1}+X_{0}^{1}X_{1}^{1}Y_{0}^{0}Y_{1}^{1}+X_{0}^{2}X_{1}^{1}Y_{0}^{1}Y_{1}^{1}+X_{0}^{2}X_{1}^{2}Y_{0}^{1}Y_{1}^{0}+X_{0}^{2}X_{1}^{2}Y_{0}^{1}\\ \; & +2Y_{0}^{0}Y_{1}^{0}\left[X_{0}^{1}+X_{0}^{2}\right]+2X_{1}^{2}Y_{0}^{0}\left[X_{0}^{1}+X_{0}^{2}\right]+2X_{1}^{1}Y_{1}^{0}+2X_{1}^{2}Y_{1}^{1}+2X_{1}^{2}Y_{1}^{0}\\ \; \end{array}$$

The negation of literals approach reduces the output $Z_{X>Y}$ to:

(27) $$\begin{array}{cc} Z_{X>Y}= & X_{0}^{2}X_{1}^{1}\bar{Y_{0}^{2}}Y_{1}^{1}+X_{0}^{1}X_{1}^{1}Y_{0}^{0}Y_{1}^{1}+X_{0}^{2}X_{1}^{2}Y_{0}^{1}Y_{1}^{0}+X_{0}^{2}X_{1}^{2}Y_{0}^{1}\\ \; & +\bar{X_{0}^{0}}X_{1}^{2}Y_{0}^{0}+\bar{X_{0}^{0}}Y_{0}^{0}Y_{1}^{0}+X_{1}^{1}Y_{1}^{0}+X_{1}^{2}\bar{Y_{1}^{2}}\\ \; \end{array}$$

## 5. Results and Discussion

This section evaluates the performance of the proposed CNTFET-RRAM-based ALU processing modules. For simulation of various ternary designs, a 32-nanometre technology node and 0.9 V supply voltage at room temperature is considered, and the analysis is performed using HSPICE simulator. For functional validation, the Stanford University CNTFET model [51] and Stanford University RRAM model [52] are implemented for analysis of all the designs. This standard model has been designed for unipolar enhancement-mode MOSFET-like CNTFET devices in which each transistor may include one or more CNTs as its channel. This model also considers a realistic, circuit-compatible CNTFET structure and includes practical device non-idealities, parasitics, Schottky barrier effects at the contacts, inter-CNT charge screening effects, doped source-drain extension regions, scattering (non-ideal near-ballistic transport), back-gate (substrate bias) effect, and source/drain and gate resistances and capacitances. The model also includes a full transcapacitance network for more accurate transient and dynamic performance simulations. Additionally, this model accounts for the parasitic contact resistance by taking into account the tunneling through the Schottky barrier at the metal-to-CNT interface. There is no dependence on the contact length. Table 5 lists parameters of the CNTFET model and their values employed in the design. The Stanford University RRAM model works on the principle of conductive filament (CF) growth between the top and bottom electrode and also takes into account critical switching phenomena, such as Joule heating and temperature change. A brief description of the RRAM parameters is given in Table 6. The simulations results obtained from the HSPICE functionally validate and authenticate the working of the proposed design modules.

### 5.1. Functional Validation

The various ternary ALU modules (adder–subtractor, multiplier, comparator, and logical modules) are validated by performing simulation using HSPICE software. The design optimization of the proposed modules is achieved by implementing designs using negation of literals technique and making use of CNTFET-RRAM-based ternary logic gates. This results in reduced component count compared to the existing designs, thereby helping to reduce the overall area of the proposed circuits.

**Figure 13:**
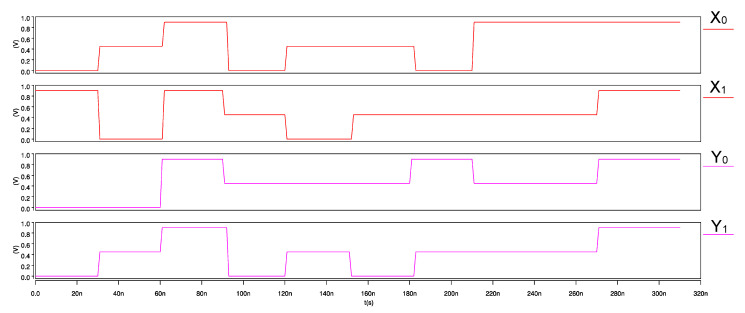
Input waveforms of CNTFET-RRAM ternary adder–subtractor module.

**Figure 14:**
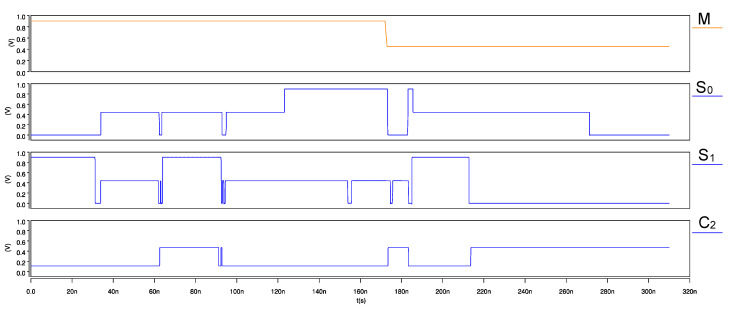
Transient response of CNTFET-RRAM ternary adder–subtractor module.

The input waveforms for the proposed ternary adder–subtractor and its transient response are shown in Figure 13 and Figure 14, respectively. On observing the simulations, we note that when the status of mode control signal M = 2, the module performs the addition operation, whereas when M = 1, the subtraction of ternary numbers is performed, thereby verifying the correct functionality of the adder–subtractor module.

**Figure 15:**
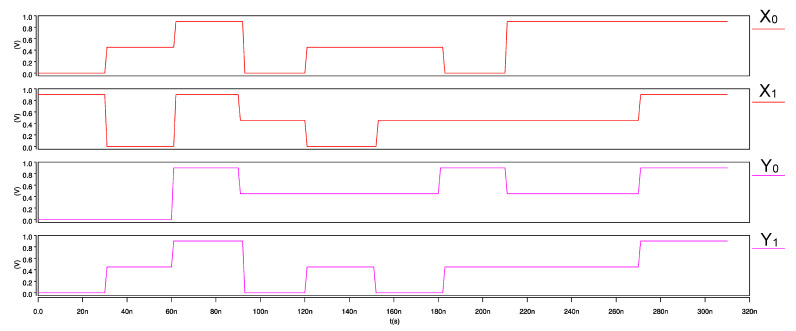
Input waveforms of 2-trit CNTFET-RRAM ternary multiplier module.

**Figure 16:**
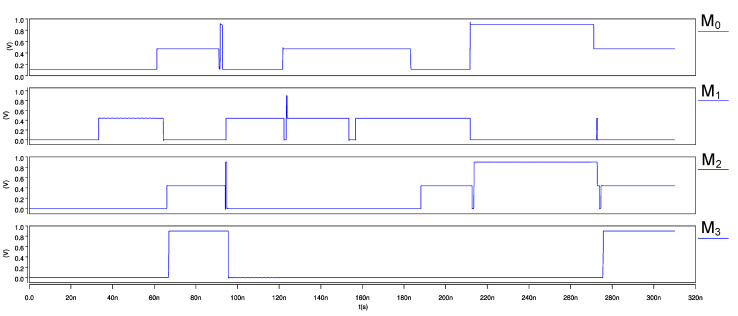
Transient response of 2-trit CNTFET-RRAM ternary multiplier module.

**Figure 17:**
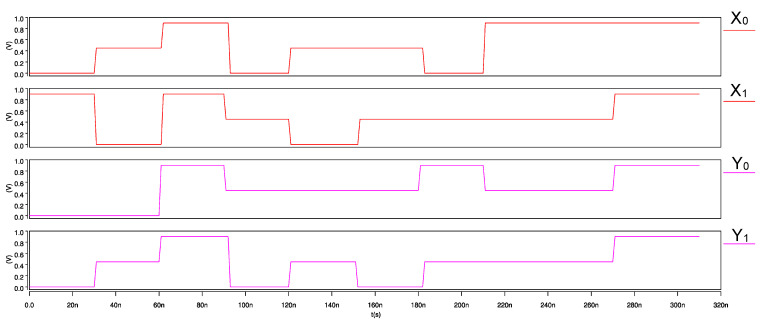
Input waveforms of 2-trit CNTFET-RRAM ternary comparator module.

**Figure 18:**
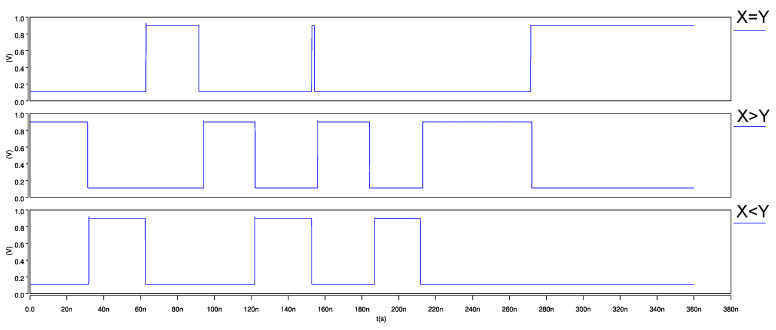
Transient response of 2-trit CNTFET-RRAM ternary comparator module.

The ternary 2-trit multiplier, input waveforms, and its transient response are shown in Figure 15 and Figure 16, respectively. Similarly, for the ternary comparator, input waveforms and its transient response are shown in Figure 17 and Figure 18, respectively.

### 5.2. Performance Comparison

**Figure 19:**
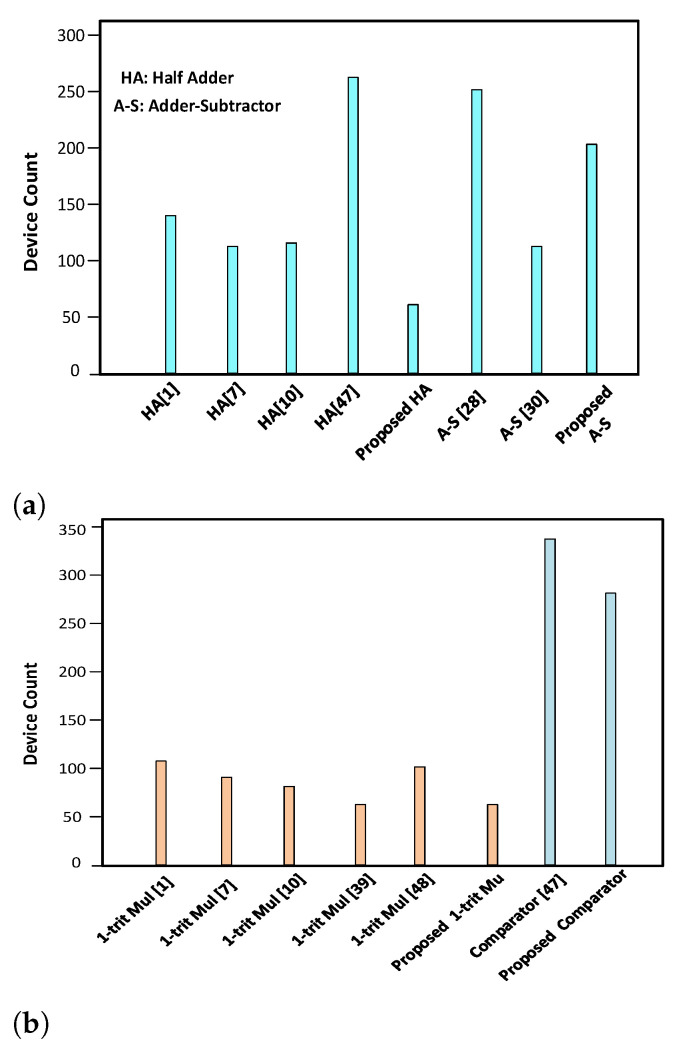
Device count comparison of arithmetic modules. (**a**) Adder designs, (**b**) multiplier and comparator designs.

For analyzing the hardware efficiency of the proposed ternary ALU modules, an investigation on the hardware requirement of the various ternary processing modules was performed. Figure 19a shows the transistor count comparison of the various adder–subtractor modules. The transistor count comparison of the multiplier and the comparator modules is shown in in Figure 19b.

The schematic of adder–subtractor module is composed of two CNTFET-RRAM ternary full adders, two CNTFET-RRAM ternary Ex-OR gates, and a PTI and STI circuit. The ternary full adder is implemented using two CNTFET-RRAM ternary half adders and a CNTFET-RRAM ternary OR gate. The ternary half adder is composed of two ternary decoders which are implemented using CNTFET-RRAM ternary logic gates, nine 2-inputs binary NAND, two 3-inputs binary NAND, two binary inverters, two 2-inputs TNAND, and one proposed level shifter. The overall transistor count of the adder–subtractor module is 199 transistors. A single ternary full adder block has 174 transistors (168 from two half adders and 8 from the OR logic gate), ternary Ex-OR gate has 20 transistors, PTI is composed of 2 transistors, whereas the STI block consists of 3 transistors.

The 2-trit multiplier module is composed of four 1-trit CNTFET-RRAM multipliers, five CNTFET-RRAM ternary half adders, and two CNTFET-RRAM ternary full adder circuits. The ternary 1-trit multiplier is composed of two ternary decoders which are implemented using CNTFET-RRAM ternary logic gates, four 2-inputs binary AND, two 2-inputs binary OR, two 2-inputs TNAND, and one 2-inputs TOR gate. The overall transistor count of the 2-trit multiplier module is 1016 transistors. A single ternary 1-trit CNTFET-RRAM multiplier has 62 transistors, each ternary half adder has 84 transistors, whereas the full adder has 174 transistors.

The comparator module is composed of four ternary decoders, ten 2-inputs binary AND, six 3-inputs binary AND, six 4-inputs binary AND, two 2-inputs binary OR, six 3-inputs binary OR, six binary inverters, a single 2-inputs TAND, and two 3-inputs TOR. The overall transistor count of the ternary comparator module is 305 transistors.

From the comparison of the transistor count for the CNTFET-RRAM ternary half adder, with the existing designs, a sizeable reduction in the transistors count is observed. The reduction in the number of transistors of ternary half adder is 38.23% compared to the ternary half adder of [1], 25% compared to [10], and 67.44% compared to [47]. Similarly, for the adder–subtractor module, the combined adder–subtractor cell of Murotiya et al. [29] utilizes 250 transistors, the design of Moaiyeri et al. [7] and Dhande and Ingole [49] uses 428 and 868 transistors, respectively, whereas the proposed CNTFET-RRAM utilizes significantly less transistors.

The comparison of power delay product (PDP) for the various ALU processing modules at three supply voltages of 0.8 V, 0.9 V, and 1 V is shown in Table 7. From the table, we observe that the PDP of the proposed CNTFET-RRAM processing modules are comparable with the various designs existing in the literature. Therefore, the integration of CNTFET-RRAM technology for the implementation of various ALU processing modules provides the advantages of the reduced hardware complexity (in terms of transistor count); in addition, the presence of RRAM makes the design non-volatile. These benefits are achieved without significantly degrading the energy consumption (PDP) of the designs.

## 6. Conclusions

This article presents the design methodology for 2-trit ternary ALU using CNTFETs and RRAM as the basic design elements. The various functional modules of the proposed ALU have been implemented and simulated using HSPICE, and the results obtained from the simulation authenticate and functionally validate the correctness of the proposed designs. The proposed ternary ALU modules are implemented, taking advantage of the variable multithreshold design method of CNTFET and multilevel cell characteristics of RRAM. On the basis of the exhaustive simulations, we deduce that the proposed designs have good robustness in addition to the important characteristic of non-volatility owing to the presence of RRAM in the design. Thus, the proposed ternary ALU can serve as an efficient processing unit for modern ternary microprocessors with CNTFETs and RRAM in the nanoscale era.

## Figures and Tables

**Table 1:** Logic symbols.

Voltage Level	Logic Value
0	0 (Low)
$\frac{V_{dd}}{2}$	1 (Intermediate)
$V_{dd}$	2 (High)

**Table 2:** Truth table of ternary inverters.

INPUT	STI	PTI	NTI
0	2	2	2
1	1	2	0
2	0	0	0

**Table 3:** Truth table of ternary NAND and ternary NOR.

INPUT $Y_{1}$	INPUT $Y_{2}$	$Z_{\mathrm{NAND}}$	$Z_{\mathrm{NOR}}$
0	0	2	2
0	1	2	1
0	2	2	0
1	0	2	1
1	1	1	1
1	2	1	0
2	0	2	0
2	1	1	0
2	2	0	0

**Table 4:** Functional table of ternary ALU.

$S_{1}$	$S_{0}$	Operation
0	0	Addition
0	1	Subtraction
0	2	Multiplication
1	0	Comparison
1	1	NAND
1	2	NOR
2	0	Ex-OR
2	1	AND
2	2	OR

**Table 5:** Characteristic parameters and their values employed for CNTFET model.

Parameter of CNTFET	Parameter Specification	Value
$L_{ch}$	Length of the physical channel	32 nm
$L_{geff}$	Mean free path length of intrinsic CNT channel	100 nm
$L_{ss}$	The length of doped CNT source-side extension region	32 nm
$L_{dd}$	The length of doped CNT drain-side extension region	32 nm
$T_{ox}$	Thickness of the top gate dielectric material	4 nm
EFI	The Fermi level of the doped S/D tube	6 ev
$K_{gate}$	The dielectric constant of high-k top gate dielectric material	16
$C_{sub}$	Coupling capacitance along the substrate and the channel	20 pF/m

**Table 6:** Characteristic parameters of RRAM model.

Parameter	Parameter Description	Value
T_ini	Initial device temperature	298 K
F_min	Minimum field to enhance gap formation	1.4 × 10${}^{9}$ V/m
$t_{ox}$	Oxide thickness	12 nm
gap_ini	Initial gap distance	1.8 nm
gap_min	Minimum gap distance	0.2 nm
gap_max	Maximum gap distance	1.8 nm
$E_{a}$	Activation energy for vacancy generation	0.6 eV

**Table 7:** Power delay product (PDP) comparison for the various ALU functional modules.

Functional Cell	PDP (fJ)	PDP (fJ)	PDP (fJ)
	at 0.8 V	at 0.9 V	at 1 V
Ternary Half Adder of Lin et al. [1]	-	0.04411	-
Ternary Half Adder of Moaiyeri et al. [7]	0.115	0.192	0.368
Ternary Half Adder of Samadhi et al. [10]	-	0.221	-
Proposed CNTFET-RRAM Ternary Half Adder	0	0.0688	0
Ternary Full Adder–Subtractor of Murotiya et al. [29]	0.380	0.634	1.218
Ternary Full Adder–Subtractor of Srivastava et al. [53]	34.9	35.5	41.2
Proposed CNTFET-RRAM Ternary Full Adder–Subtractor	73.1	87.3	90.03
Ternary 1-trit Multiplier of Lin et al. [1]	-	0.248	-
Ternary 1-trit Multiplier of Sridharan et al. [9]	-	0.164	-
Proposed CNTFET-RRAM Ternary 1-trit Multiplier	0.1011	0.1587	0.2283

## Abbreviations

The following abbreviations are used in this manuscript:

RRAM	Resistive Random Access Memory
CNTFET	Carbon Nanotube Field Efect Transistors
STI	Standard Ternary Inverter
NTI	Negative Ternary Inverter
PTI	Positive Ternary Inverter
ALU	Arithmetic Logic Unit
